# Preparing for the New Normal: Chronicling the Impact of COVID-19 on Older Adults and Providers

**DOI:** 10.1093/geroni/igab046.2236

**Published:** 2021-12-17

**Authors:** Rose Ann DiMaria-Ghalili, Justine Sefcik

## Abstract

COVID-19 and social distancing heralded an unprecedented change in the way older adults and health care providers live, work, socialize and manage their health. Early “calls-to-action” included the call for researchers to chronicle the impact of the COVID-19 pandemic on care of older adults to inform models of care and best practices in the new normal. This symposium explores the impact of COVID-19 on the health of older adults across the care continuum and healthcare delivery augmented by technology. The perspectives of older adults living in the community and providers who care for this population are highlighted. Additionally, there is a focus on the most vulnerable, those living in skilled care facilities and continuing care retirement communities. Fisher analyzes the key themes in 37 COVID-19 video communiques over 11 months at a continuing care retirement community. Sefcik explores coping strategies including outdoor activities among community-dwelling older adults. DiMaria-Ghalili examined patterns of physical and mental health, technology usage and loneliness in older adults, including those living in the community and a continuing care retirement community. Using longitudinal data and COVID-19 supplemental survey data from the National Health and Aging Trends Study, Huh-Yoo discusses disparities in online patient-provider communication and implications for the Post-COVID era. Coates discusses the facilitators and barriers perceived by interdisciplinary providers deploying telehealth during the COVID-19 pandemic and implications for healthcare delivery in older adults. The symposium will conclude with a discussion by Dr. Sefcik on the implications for research, practice and policy in the post COVID-19 era.

